# Cowden Syndrome: A Case Series Highlighting Cutaneous and Systemic Diversity

**DOI:** 10.7759/cureus.67241

**Published:** 2024-08-19

**Authors:** Marcus S Rossi, Jessica P Sejo, Allison Kirchner, Maria Tsoukas

**Affiliations:** 1 Department of Dermatology, University of Illinois College of Medicine, Peoria, USA; 2 Department of Dermatology, University of Illinois Chicago College of Medicine, Chicago, USA

**Keywords:** skin neoplasm, pten mutation, phosphatase and tensin homolog, genodermatoses, cowden syndrome

## Abstract

Cowden syndrome (CS) is an autosomal dominant genetic disorder characterized by multiple hamartomas across various tissues and an elevated risk of several types of cancer, including breast, thyroid, and endometrial cancers. Skin findings can precede more serious malignancies, making early detection and diagnosis crucial. In this report, we detail four individual patient histories, including their initial dermatological symptoms or concerns. Due to the wide variety of their clinical presentations, this report highlights the variable level of symptom severity in the presentation of CS and how this may lead to a challenging diagnosis.

## Introduction

Cowden syndrome (CS) affects approximately 1 in 200,000 people worldwide, often linked to mutations in the PTEN gene, located on chromosome 10q23 [1]. Cutaneous manifestations, often the first noticeable signs of CS, range from facial papules and acral keratoses to atypical lesions like lipomas and neurinomas [2-5]. It is a rare autosomal dominant condition associated with hamartomas of ectodermal, mesodermal, and endodermal origin and the increased risk of breast, thyroid, and potentially other cancers such as renal, colorectal, or endometrial [6]. Many patients show a germline mutation in the tumor suppressor gene, phosphatase and tensin homolog (PTEN) [7].

The PTEN gene for CS has been mapped by linkage analysis to a 6 cM region on the long arm of chromosome 10 at 10q22-23 [1,8]. This gene, located on chromosome 10q23, is composed of 9 exons that encode a predominant protein product of 403 amino acids, 48 kDa, and shares sequence homology with the tyrosine phosphatase superfamily, tensin, and auxilin [8]. PTEN is a gene that produces a protein, which functions as a tumor suppressor by lipid phosphatase activity, which regulates the PI3Ks pathway [8]. When the function of the gene is lost, there is no regulation of the PI3Ks pathway, leading to increased cell survival and proliferation [8]. In families affected by CS, PTEN mutations are transmitted in an autosomal dominant manner, giving each child of an affected individual a 50% chance of inheriting the syndrome. Remarkably, up to 45% of CS cases arise from new (de novo) PTEN mutations, and a smaller fraction from mosaicism-where the mutation is present in some but not all cells of a parent, which may have been previously mistaken for de novo mutations [2].

A notable proportion of individuals who meet the clinical criteria for CS do not harbor detectable PTEN mutations, suggesting alternative genetic underpinnings [2]. Some patients diagnosed with CS have germline mutations in the SDHB, SDHC, and SDHD genes, typically associated with hereditary pheochromocytoma and paraganglioma syndrome, sharing a predisposition to thyroid and renal cancers seen in CS [2]. Furthermore, the hypermethylation of the KILLIN promoter, which shares regulatory sequences with PTEN, has been observed in some CS cases, pointing to a potential epigenetic dimension in CS pathogenesis [2].

In CS, cutaneous manifestations are present in 90 to 100% of cases and often serve as the initial indicators of the disease [3,5]. The most common cutaneous findings are the presence of numerous verrucous lesions [9,10]. Papillomas, similar in distribution but larger, are the second most frequent lesions [11]. Other typical features include small, multiple facial papules ranging from 1 to 4 mm in diameter with smooth or keratotic surfaces, commonly located on the eyelids, forehead, nose, around the mouth, and occasionally on the auricular pavilions. Additionally, acral keratoses, rough keratotic papules on the dorsal sides of forearms, hands, and feet, are also characteristic [12]. Translucent keratotic papules, small, indurated lesions sometimes centered by a depression, are typically found on palms and soles [4,12].

Beyond the above more common lesions, CS may present with non-typical cutaneous lesions including benign tumors like angiomas, dermal fibromas, and neurofibromas, as well as malignant tumors such as melanomas and basal cell carcinomas [3,11]. Dyschromic lesions, including "café au lait spots" and vitiligo, are also observed [5]. Taken together, this highlights the wide variety of cutaneous manifestations attributed to CS. 

CS can be a challenging diagnosis to make due to the variability of the cutaneous findings. Although difficult, a swift diagnosis of CS by a dermatologist is paramount, as mucocutaneous lesions often precede the development of malignancies. Therefore, to prevent or detect cancer early, a quick confirmation of a CS diagnosis is imperative, followed by proper referrals [2,9,10].

In this article, we describe a series of four patients who presented to a United States teaching hospital. Each patient has a genetically proven diagnosis of Cowden Syndrome and demonstrated unique cutaneous, extracutaneous, and genetic manifestations, highlighting the variable, but at times predictable, findings of this disorder.

## Case presentation

Case 1

A 44-year-old female presented to the dermatology clinic for evaluation of long-standing facial lesions, which she reported having for many years. These lesions remained stable without any associated discomfort or symptomatic changes in size, shape, or appearance. Upon examination, she was found to have three flesh-colored verrucous papules on the chin and left cheek, each measuring 5-6 mm. Based on clinical appearance, these lesions were identified as trichilemmomas.

In addition to her dermatological presentation, the patient had a significant medical history of dysplastic gangliocytoma, for which she had undergone surgical resections twice, once in 2004 and again in 2018. Furthermore, she had a history of endometrial hyperplasia that was managed with a total abdominal hysterectomy in 2017. This patient also had a thyroidectomy performed in 2017, along with a history of multiple GI polyps identified by colonoscopy. The patient also underwent genetic testing, with results showing she was positive for a deleterious mutation in PTEN (EX6del), proving a diagnosis of CS.

Case 2

An 11-year-old male presented for evaluation of a tumor on his right leg, which was first noticed by his pediatrician two years prior. His mother reports that the mass had increased in size over the past month, although there have been no other symptoms associated with this change. On physical examination, the mass was characterized as a 4 × 3 cm soft, mobile subcutaneous nodule on the right shin, consistent with a diagnosis of lipoma. Further examination revealed additional noteworthy findings: on the left first digit, a glossy domed 3 mm papule at the base was diagnosed as an acral neural hamartoma, and a similar lesion, a glossy domed 4 mm papule, was present on the right third distal dorsal digit. In the oral cavity, hypertrophic papules were observed between the teeth on the gingiva, diagnosed as mucosal papilloma.

The patient's medical history is significant for multiple thyroid nodules for which he underwent thyroidectomy. The patient also underwent genetic testing and is heterozygous for a known PTEN mutation (c.493-2A>G), proving a diagnosis of CS.

Case 3

**Figure 1:**
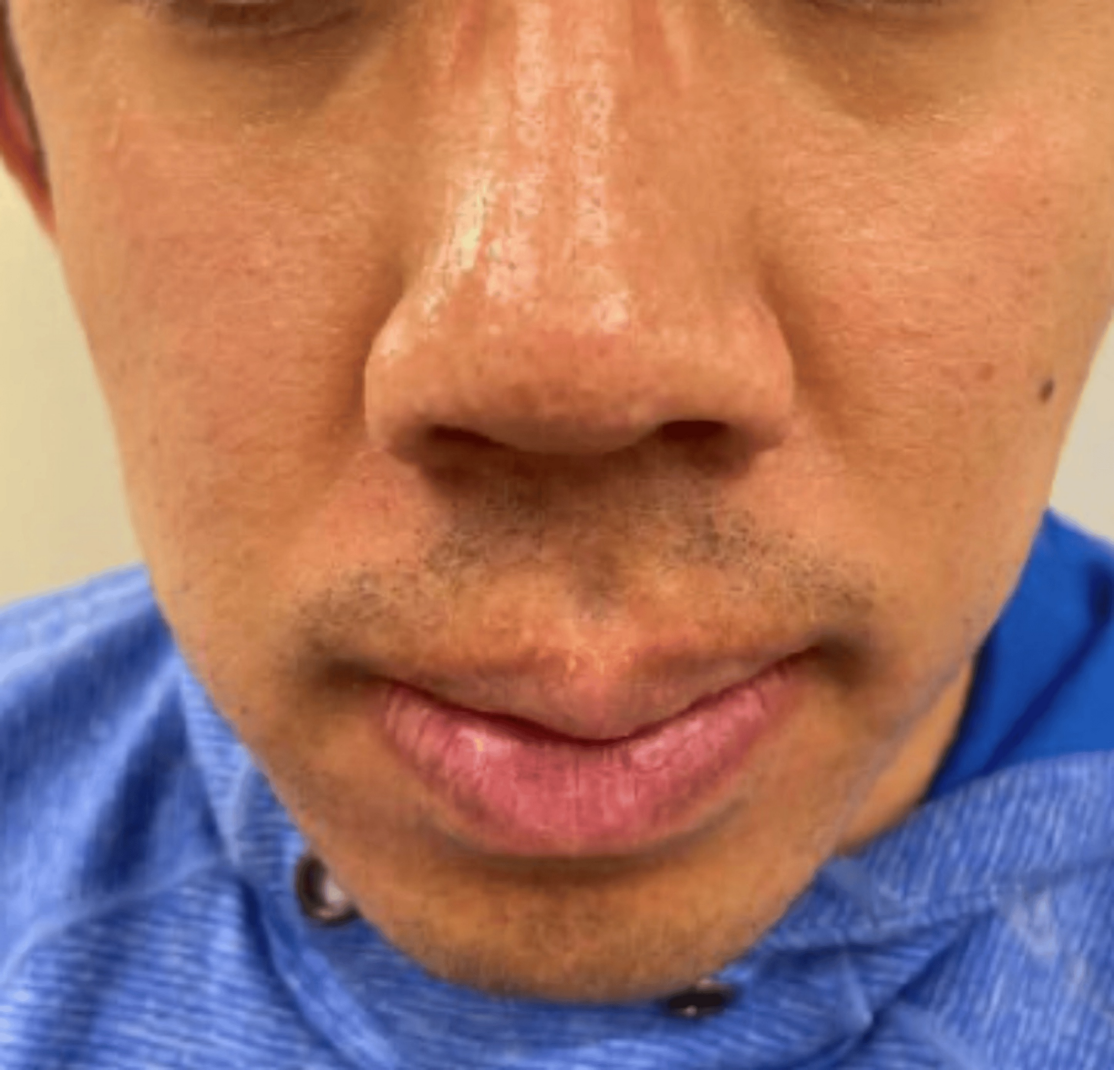
Numerous skin-colored papules of 1-3 mm diameter (trichilemmomas)

**Figure 2:**
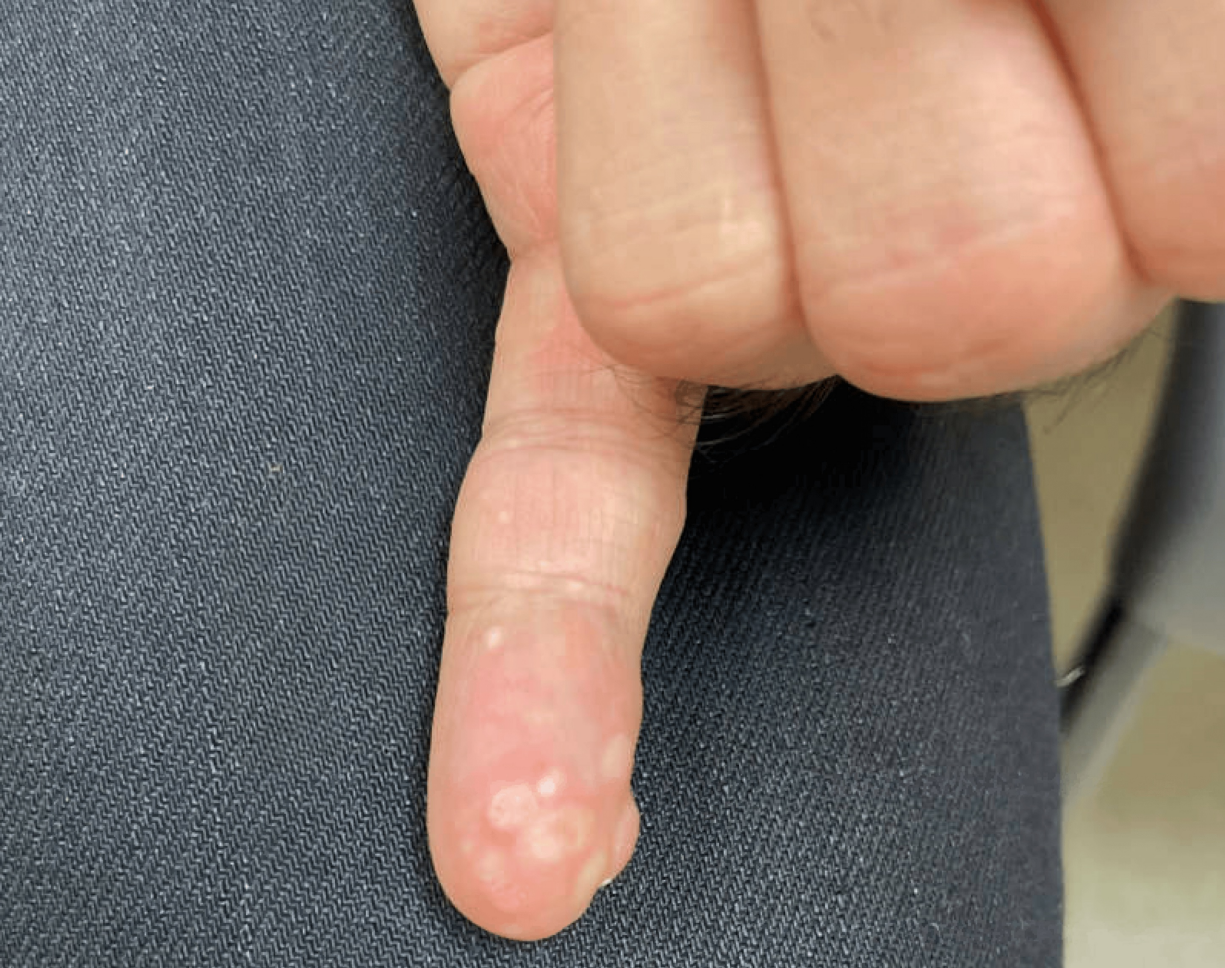
Translucent keratotic papules over the distal left fifth finger (acral keratosis)

A 34-year-old male sought evaluation for longstanding lesions on his back, fingers, and face. These lesions, first noted in childhood and of unknown pathology, were asymptomatic but became a cosmetic concern for the patient. Upon examination, numerous skin-colored papules of 1-3 mm diameter were identified on the patient's nose, consistent with a diagnosis of trichilemmomas (Figure 1). Additionally, a flesh-colored plaque measuring 1.2 × 1.0 cm was noted on the right lower back, which was surgically excised and was histologically consistent with neurofibroma. His hands displayed translucent keratotic papules over the distal left fifth finger, suggestive of acral keratosis, also known as palmoplantar translucent keratosis (Figure 2). Multiple mucosal papillomas were also present during the physical examination.

In exploring the patient’s family history, a pattern suggestive of CS began to emerge. His father had similar skin findings, while his paternal aunt had a history of thyroid and endometrial cancer, and his brother also shared similar skin lesions. The patient himself also tested positive for a deleterious mutation in PTEN (p.R335* (c.1003C>T). This proved him to have a diagnosis of PTEN hamartoma tumor syndrome - CS.

Case 4

A 32-year-old female presented, with multiple tan macules and patches on her bilateral plantar feet and several papules scattered across the forehead and nasal ala, diagnosed as mild trichilemmomas. Additionally, plantar hyperkeratotic pits were observed.

From an oncological perspective, the patient has a significant medical history of a right breast malignant phyllodes tumor with sarcomatous features treated like a breast sarcoma. Post-treatment, she had a right-modified radical mastectomy, followed by adjuvant chemotherapy and chest wall radiotherapy. She remained free from recurrence. However, a diagnosis of atypical ductal hyperplasia in the left breast led to the commencement of chemo-preventive tamoxifen therapy. She has also undergone a total thyroidectomy. Evaluation by a genetic counselor with germline testing showed she was positive for a pathogenic mutation in the PTEN gene (p.K332* (c.994A>T)) and the LZTR1 gene (c.1149+1G>A), proving a diagnosis of CS.

## Discussion

CS is a rare autosomal dominant disorder that presents with a broad spectrum of cutaneous and extracutaneous manifestations [1,8]. The four cases presented in this series illustrate the diverse clinical presentations and variable grade of symptom severity of CS, summarized in Table 1, and highlight the importance of thorough dermatological and genetic evaluations.

**Table 1:** Summary of extracutaneous and genetic findings in four patients diagnosed with Cowden syndrome

Case	Age	Sex	Extracutaneous findings	Uncommon cutaneous findings	Genetic findings
1	44	F	Dysplastic gangliocytoma; endometrial hyperplasia; thyroidectomy; GI polyps	None	Positive for a deleterious mutation in PTEN (EX6del).
2	11	M	Thyroid nodules; thyroidectomy	None	Heterozygous for a known PTEN mutation (c.493-2A>G).
3	34	M	None	Neurofibroma	Family history (+) for thyroid cancer; endometrial cancer. Personal history (+) for deleterious mutation in PTEN (p.R335* (c.1003C>T).
4	32	F	Malignant phyllodes tumor; atypical ductal hyperplasia; thyroidectomy	None	Positive for a pathogenic mutation in the PTEN gene (p.K332* (c.994A>T) and the LZTR1 gene (c.1149+1G>A).

Cutaneous manifestations are often the first signs of CS and play a crucial role in early diagnosis, with previous reports emphasizing that cutaneous lesions are present in 90-100% of CS cases and often precede malignancies [2-5,9].

Table 2 highlights the prevalence of common cutaneous findings in our cohort of four patients diagnosed with CS, comparing these findings against historical data from a comprehensive review by Lim et al. [13]. These findings were consistent with known presentations of the syndrome but also highlighted some variability. Trichilemmomas were present in 75% of patients, slightly below the historical range of 90-100%. Acral keratosis was also noted in 75% of patients, aligning with the historical prevalence of 63-73%. Lipomas and mucosal papillomas were observed in 25% and 50% of cases, respectively, lower than the historical frequencies of 34-56% and 83-86%.

**Table 2:** Summary of common cutaneous manifestations of four patients diagnosed with Cowden syndrome in comparison to the historical average

Cutaneous finding	Case 1	Case 2	Case 3	Case 4	Present (%)	Historical (%)^13^
Trichilemmomas	Yes	No	Yes	Yes	75	90-100
Acral keratosis	No	Yes	Yes	Yes	75	63-73
Lipomas	No	Yes	No	No	25	34-56
Mucosal papillomas	No	Yes	Yes	No	50	83-86

Extracutaneous findings were significant in all four patients, demonstrating the multisystem involvement typical of CS. Patient 1's history of dysplastic gangliocytoma and endometrial hyperplasia, Patient 2’s thyroid nodules with a history of thyroidectomy, and Patient 4's complex oncologic history, including a malignant phyllodes tumor and atypical ductal hyperplasia, highlight the syndrome's broad impact. These cases underline the necessity for coordinated multidisciplinary care involving oncology, surgery, and dermatology to manage CS effectively [6,11].

Family history played a crucial role in diagnosing CS, particularly in Patient 3, whose familial patterns of similar cutaneous findings and malignancies provided critical diagnostic clues. This pattern is consistent with the autosomal dominant inheritance of PTEN mutations, which are present in approximately 80% of CS cases [1,8]. Moreover, up to 45% of CS cases arise from de novo PTEN mutations, further complicating the genetic landscape [2].

Genetic testing was pivotal in confirming CS diagnoses in our case series. All four patients tested positive for pathogenic variants in the PTEN gene (described in Table 1), confirming the clinical suspicion of a CS diagnosis. Additionally, the identification of a pathogenic variant in LZTR1 in Patient 4 underscores the genetic heterogeneity within CS and the potential overlap with other heritable cancer syndromes [2].

## Conclusions

In conclusion, our case series reinforces the critical role of dermatologic evaluation in the early recognition of CS. The interplay between cutaneous and systemic manifestations, along with relevant family history, necessitates a multidisciplinary approach to care. Genetic counseling and vigilant surveillance for malignancies are essential in managing CS and optimizing patient outcomes in this complex hereditary syndrome. Early detection and diagnosis can significantly impact the management and prognosis of patients with CS, highlighting the need for heightened awareness and comprehensive evaluation protocols among healthcare providers.

## References

[1] Marsh DJ, Dahia PL, Coulon V (1998). Allelic imbalance, including deletion of PTEN/MMACI, at the Cowden disease locus on 10q22-23, in hamartomas from patients with Cowden syndrome and germline PTEN mutation. Genes Chromosomes Cancer.

[2] Gammon A, Jasperson K, Champine M (2016). Genetic basis of Cowden syndrome and its implications for clinical practice and risk management. Appl Clin Genet.

[3] Kacem M, Zili J, Zakhama A, Hadj Youssef F, Mahjoub S, Boubakri C, El May M (2000). Multinodular goiter and parotid carcinoma: a new case of Cowden's disease. Ann Endocrinol (Paris).

[4] Botma M, Russell DI, Kell RA (2002). Cowden's disease: a rare cause of oral papillomatosis. J Laryngol Otol.

[5] Hildenbrand C, Burgdorf WH, Lautenschlager S (2001). Cowden syndrome-diagnostic skin signs. Dermatology.

[6] Pilarski R (2009). Cowden syndrome: a critical review of the clinical literature. J Genet Couns.

[7] Ha JW (2013). Autosomal dominant inherited Cowden's disease in a family. Clin Endosc.

[8] Álvarez-Garcia V, Tawil Y, Wise HM, Leslie NR (2019). Mechanisms of PTEN loss in cancer: it's all about diversity. Semin Cancer Biol.

[9] Arai H, Akagi K, Nakagawa A, Onai Y, Utsu Y, Masuda S, Aotsuka N (2023). Clinical and genetic diagnosis of Cowden syndrome: a case report of a rare PTEN germline variant and diverse clinical presentation. Medicine (Baltimore).

[10] Magaña M, Landeta-Sa AP, López-Flores Y (2022). Cowden disease: a review. Am J Dermatopathol.

[11] Boutet G, Boisserie-Lacroix M (1995). Cowden's disease in a young girl: mammographic problems. J Gynecol Obstet Biol Reprod (Paris).

[12] Longy M, Lacombe D (1996). Cowden disease. Report of a family and review. Ann Genet.

[13] Lim A, Ngeow J (2021). The skin in Cowden syndrome. Front Med (Lausanne).

